# The Impact of Immunological Checkpoint Inhibitors and Targeted Therapy on Chronic Pruritus in Cancer Patients

**DOI:** 10.3390/biomedicines9010002

**Published:** 2020-12-22

**Authors:** Alessandro Allegra, Eleonora Di Salvo, Marco Casciaro, Caterina Musolino, Giovanni Pioggia, Sebastiano Gangemi

**Affiliations:** 1 Division of Hematology, Department of Human Pathology in Adulthood and Childhood “Gaetano Barresi”, University of Messina, 98125 Messina, Italy; cmusolino@unime.it; 2 Department of Veterinary Sciences, University of Messina, 98125 Messina, Italy; eleonora.disalvo6@gmail.com; 3 School of Allergy and Clinical Immunology, Department of Clinical and Experimental Medicine, University of Messina, 98125 Messina, Italy; marco.casciaro@unime.it (M.C.); gangemis@unime.it (S.G.); 4 Operative Unit of Allergy and Clinical Immunology, Department of Clinical and Experimental Medicine, University of Messina, 98125 Messina, Italy; 5 Institute for Biomedical Research and Innovation (IRIB), National Research Council of Italy (CNR), 98164 Messina, Italy; giovanni.pioggia@cnr.it

**Keywords:** pruritus, cancer, skin, adverse drug reaction, chemotherapy, immunological checkpoint inhibitors, target therapy, tyrosine kinase inhibitors, monoclonal antibodies

## Abstract

Although pruritus may sometimes be a consequential situation to neoplasms, it more frequently emerges after commencing chemotherapy. In this review, we present our analysis of the chemotherapy treatments that most often induce skin changes and itching. After discussing conventional chemotherapies capable of inducing pruritus, we present our evaluation of new drugs such as immunological checkpoint inhibitors (ICIs), tyrosine kinase inhibitors, and monoclonal antibodies. Although ICIs and targeted therapy are thought to damage tumor cells, these therapies can modify homeostatic events of the epidermis and dermis, causing the occurrence of cutaneous toxicities in treated subjects. In the face of greater efficacy, greater skin toxicity has been reported for most of these drugs. A remarkable aspect of some reports is the presence of a probable correlation between cutaneous toxicity and treatment effectiveness in tumor patients who were treated with novel drugs such as nivolumab or pembrolizumab. Findings from these experiments demonstrate that the occurrence of any grade of skin side effects can be considered as a predictor of a better outcome. In the near future, studies on the relationship between the onset of skin alterations and outcomes could open new perspectives on the treatment of neoplasms through specific target therapy.

## 1. Introduction

### 1.1. General Considerations on Pruritus

Pruritus is an unlikable sensation that provokes a wish to scratch, in response to mechanical, chemical, or thermal motivations. This condition is due to several systemic or dermatological diseases or neurologic and autoimmune pathologies. As far the mechanisms of pruritus mediation and modulation, pruritus is stimulated and regulated by different exogenous or endogenous pruritogens and their receptors. Pruritus is classified into four diverse clinical groups. These are systemic, neuropathic, psychogenic, and pruritoceptive [1]. The molecular systems implicated in pruritus sensation are extremely complicated and remain indefinable in most of these conditions, as an enormous quantity of receptors, mediators, and controllers responsible for pruritus have been detected [2]. The most well-recognized distinction between forms of pruritus is that of histaminergic and non-histaminergic pruritus [3]. Acute itch is controlled through both pathways [4,5,6]. In contrast, chronic itch is essentially regulated by the non-histaminergic pathway [6]. The histaminergic system stimulates the transient receptor potential vanilloid 1 (TRPV1) channel while the nonhistaminergic system stimulates TRPV1 or transient receptor potential ankyrin 1 (TRPA1) [7]. In both systems, histaminergic and nonhistaminergic, TRPV1/TRPA1 stimulates NaV1.7, and successively, NaV1.7 regulates action potentials in neurons [8,9].

The greater part of itch receptors are components of the class A G protein-coupled receptors (GPCR). GPCRs are the principal group of membrane receptors discovered in eukaryotes. To date, about 35% of all drugs affect diverse classes of GPCRs [10,11].

### 1.2. Cancer and Pruritus

Pruritus is often a non-specific symptom of a manifest or occult neoplasm. Although this is most frequently reported with hematological malignancies, it is also described with several types of solid cancers such as those deriving from the liver, gastrointestinal system, and breast. In reports of subjects with non-specific generalized pruritus, the underlying neoplasm was reported to be the origin of itch in about 10% of subjects [12]. The relationship between cancer and pruritus has yet to be clarified; however, several mediators have been proposed to have a function. Recent findings suggest that the T-cell alterations present in Hodgkin’s lymphoma patients participate in the onset of pruritus correlated with this neoplasm and the cytokines interleukin (IL)-6, IL-8, and IL-31 may also have a part in chronic itch [13].

Nevertheless, although pruritus may sometimes be a consequential situation to the neoplasms, it more frequently emerges after commencing chemotherapy. Tumor treatment is distinguished by a great occurrence of side effects, and serious unfavorable events may alter patients’ quality of life (QOL) [14]. In a recent report, findings from more than a thousand subjects treated with about five thousand chemotherapy cycles were examined. Remarkably, among the side effects considerably associated with a reduced EuroQol 5 Dimension 5 Level (EQ-5D-5L) utility value were pruritus, and dry skin [15]; however, the effect of chemotherapy-induced pruritus on the neoplastic subject may be even more significant. One study stated that about 20–30% of subjects experiencing anti-tumor chemotherapy suffer from pruritus [16], and in these subjects, pruritus could modify not only the QoL but also the effects of anti-tumor treatment, as grave pruritus caused by chemotherapy would necessitate dosage adjustment or even suspension of the anti-tumor drugs [16,17,18].

As far as the mechanisms by which chemotherapy can induce pruritus, several hypotheses have been formulated. Unspecific cytotoxic actions on the skin provoked by the drugs or by their metabolites are the most frequent occurrences and can be detected in up to 30% of all tumor subjects, irrespective of the nature of primary cancer, but reliant on the schedule and the protocol of chemotherapy. Moreover, personal pathologic elements such as comorbidities, concomitant drugs, cutaneous alterations, pharmacogenetics, and genetic predisposition seem to be relevant [19]. Furthermore, the discharge of cytostatics or their derivatives by eccrine sweat can provoke both direct deleterious actions owed to accretion in the stratum corneum, and inflammatory events due to the reduction of the antioxidative ability of the skin [20]. Moreover, alterations of growth and differentiation of interfollicular keratinocytes and epidermal stem cells can be detected [21].

The pathogenesis of drug-caused pruritus changes depending upon the causal agent. Pruritus may be consequent to drug-provoked skin lesions, but several other possible mechanisms have been proposed, comprising phototoxicity, xerosis of the skin, or accumulations of drugs or their metabolites in the skin. However, often, the primary mechanism is not identified [22] (Table 1).

For instance, an increase of mast cells in the lesional skin of subjects treated with erlotinib might be responsible for the onset of pruritus; inhibition of degranulation of mast cells by aprepitant might explain the antipruritic action of the NK1R antagonist in these subjects [46]. Moreover, the dermatologic reactions could be due to the Epidermal Growth Factor Receptor (EGFR) inhibition in epidermal and follicular keratinocytes, able to induce modifications in keratinocyte growth, differentiation, migration, and attachment [39]. Subsequently, inflammatory cells delivery of chemoattractant factors that result in leukocyte recruitment and the release of enzymes and levels of interleukin-1, tumor necrosis factor-alfa, and IL-8 cytokines may lead to pruritus [47,48].

A similar mechanism could also be envisaged as regards the onset of pruritus after administration of immune checkpoint inhibitors, able to cause an increase of immune cytokines that may cause skin toxicities.

However, other mechanisms could be hypothesized for other drugs, and the occurrence of pruritus after paclitaxel administration appears to be secondary to skin lesions or to other unknown effects [23,24,25,26].

Finally, several drugs such as lapatinib are reported to cause chronic pruritus by unidentified mechanisms. In this group of drug-induced pruritus, therapy is very difficult, including the decision to interrupt or change the drug prescription. According to general experience, interruption for at least 6 weeks is necessary to prove that chronic pruritus is due to the suspect drug [53].

In any case, the normal course of drug-caused itch is determined by the drug administered. Drug-caused pruritus may be acute with a duration of only some days or chronic, lasting for weeks or months. It may commence with the first dispensation or may be deferred in time. For instance, a diagnosis of a deferred hypersensitivity reaction to chlorambucil was described [30].

For some substances, a clear time-relation has been reported and stoppage of the drug causes cessation of pruritus. Itch generally persists less than 6 weeks in this group, satisfying the definition of acute pruritus. In other cases, pruritus persists longer due to the different primary mechanisms. For example, in some forms of drug-caused pruritus, neuronal storing of the drug provokes pruritus, which gradually decreases after degradation of the drug. This can be grouped as chronic pruritus since it persists for more than 6 weeks.

In general, pruritic drug reactions due to the novel class of antineoplastic substances such as epidermal growth factor receptor inhibitors cause acute pruritus [54], although after pembrolizumab it may continue for numerous months after the suspension of therapy.

Many other cases of chemotherapy-provoked itch fall into the acute pruritus group. For example, as for paclitaxel, itch begins after one to three cycles, with onset 1–14 days after paclitaxel administration. Generally, duration of pruritus is 3–14 days [24,25].

Similarly, after lapatinib administration, skin collateral effects tended to be recognized early after administration and the median duration was 29 days, while subjects with Policytemia Vera (PV) experienced cutaneous side effects during the first month of pegIFN-alfa treatment in a clinical experiment [49]. Finally, most of the skin toxicity from BRAF-i therapy occurs between weeks 8 and 36 of the treatment [55].

In this review, we present our analysis of the chemotherapy treatments that most often induce skin changes and itching. After having briefly mentioned conventional chemotherapies capable of inducing itching, we discuss new drugs such as immunological checkpoint inhibitors and drugs used in so-called targeted therapy such as tyrosine kinase inhibitors and monoclonal antibodies.

## 2. Conventional Chemotherapy and Pruritus

Breast tumors are the most frequent neoplasm malignancy found in women. Several drugs implicated in the therapy of breast cancer can cause pruritus and skin alterations have been a permanent problem in the therapy of this tumor. Some of the most employed antitumor treatments, e.g., taxanes, pegylated liposomal doxorubicin, and capecitabine, provoke relevant skin alterations. The gravity and severity of these dermatologic toxicities are mainly responsible for initially employing minor dosages, e.g., for capecitabine [56].

However, some new formulations utilized to cure breast tumors can also provoke pruritus. Nanotechnology is a new area of research that has grown quickly [57]. Nab-paclitaxel is a new, albumin-bound, 130 nm particle preparation of paclitaxel, which is discharged in a suspension of albumin particles [58]. As reported in a phase II study, nab-paclitaxel was efficacious for Chinese breast cancer subjects with metastatic tumors [59]; however, it was observed that, in contrast with subjects cured with sb-paclitaxel, those cured with nab-paclitaxel displayed pruritus more often (9% vs. 27%). This rate was greater than those described in western nations [27,28].

In a different report, Thang et al. assessed the effectiveness and security of the administration of nab-paclitaxel and cisplatin in women with metastatic breast cancer. In this case, also, the occurrence of skin alterations was reported to be greater than what was described for western patients [29]. The albumin constituent of nab-paclitaxel might be the origin of the skin alterations [29].

Another drug used in the treatment of breast cancer is interferon (IFN). Partially purified human beta interferon (HuIFN-beta) was given to subjects with metastatic breast carcinoma, and pruritus attributable to the IFN happened in all patients [60].

A different form of interferon is the pegylated interferon alpha-2b (pegIFN alpha-2b), a recombinant interferon alpha-2b, which is covalently bound to polyethylene glycol (PEG). PegIFN alpha-2b has been employed in various malignancies, such as leukemias. During treatment, cutaneous side effects happen for up to 10% of the subjects, especially pruritus, erythema, cutaneous xerosis, and exanthems [61].

However, interferon alpha is used in other forms of neoplasms. During experiments on subjects with metastatic melanoma treated with pegIFN alpha-2b (SoraPeg study) and sorafenib, several and grave cutaneous alterations were reported. In fact, 24.4 % of subjects that presented with pruritus also present with exanthems, hand–foot syndrome, and alopecia. Due to the cutaneous symptoms, dose reductions or interruptions of treatment were made in several patients. The combined treatment with sorafenib/pegIFN alpha-2b provoked more cutaneous side effects than have been described for single drugs [62].

Finally, numerous hematological neoplasms are accompanied by significant itching and this symptom can be aggravated by drugs such as cytarabine and bleomycin.

Cytarabine is a pyrimidine antagonist generally employed in the therapy of hematologic malignancies such as leukemia and non-Hodgkin’s lymphoma [63,64,65]. High dosages provoke cutaneous toxicity with a reported incidence varying from 2% to 72% [66,67,68].

Several theories explaining the cutaneous alterations induced by cytarabine have been suggested, referring to immune reactions, or direct epithelial toxicity [31,32,33,34]. Furthermore, an altered process of maturation of keratinocytes as an effect of chemotherapy with cytarabine is frequently reported in the literature [35,36].

Bleomycin is a different drug employed in hematologic malignancies [69]. It is a chemotherapeutic antibiotic and it acts by blocking DNA uptake of thymidine in the S-phase of the cell cycle. It has been utilized in the treatment of Hodgkin’s lymphoma and for some germ cell tumors. Numerous cutaneous reactions to bleomycin are reported in the literature, with a percentage ranging from 8% to 20% in subjects getting a total dosage of >100 units [70,71].

## 3. Immune Checkpoint Inhibitors, Targeted Therapies, and Pruritus

**Figure 1:**
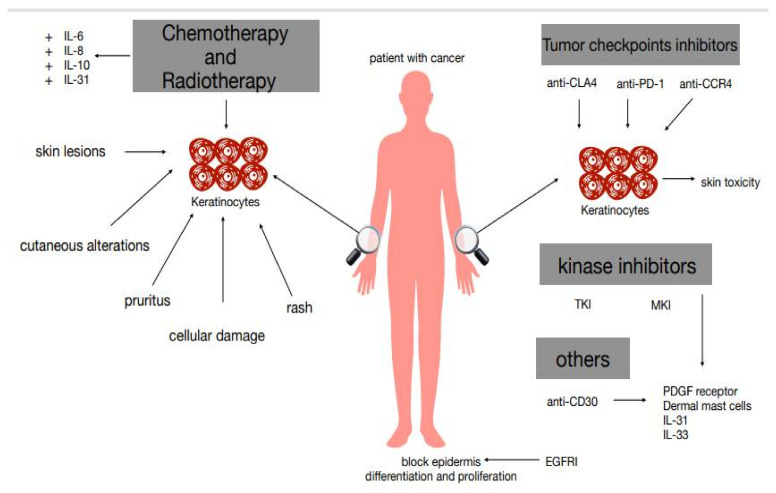
Possible mechanisms of cutaneous pruritus happening during cancer treatment.

Novel anti-tumor treatments comprising immune checkpoint inhibitors (ICIs) and targeted therapies are conceived to target alterations in the immune system and defects in DNA repair pathways and to aim at specific tumor cells. However, these therapies alter signaling pathways present in both malignant cells and normal cells and can modify homeostatic events of the epidermis and dermis; therefore, although planned to cure malignancies, targeted therapies and immunotherapies also alter the skin and its appendages, causing the occurrence of cutaneous toxicities in practically all treated subjects. In the face of greater efficacy, greater skin toxicity has been reported for most of the new drugs that we examine in the following paragraphs (Figure 1).

Antibodies against programmed cell death 1 (PD-1), a checkpoint in the effector phase of cytotoxic T cells, have been efficaciously employed in tumor immunotherapy. PD-1 blocks T-cell–mediated immune responses by attaching to its ligands, specifically PD-L1 and PD-L2. By avoiding the binding of PD-L to PD-1, some antibodies, such as pembrolizumab and nivolumab, stimulate T-cell–mediated cytotoxic activities, which determine cancer improvement in a multiplicity of tumors. Since 2017, anti-PD-1/PD-L1 antibodies have been employed to cure melanoma, cutaneous squamous cell carcinoma, lymphoma, gastric cancer, liver cancer, Merkel cell carcinoma, and other diseases [72,73,74], demonstrating superior overall response rates (ORR) and progression-free survival (PFS) compared to conventional chemotherapy [75,76,77,78].

Adjuvant immune checkpoint blockade with anti-cytotoxic T-lymphocyte-associated protein 4 (anti-CTLA-4) antibody ipilimumab is a different type of inhibition and shows an increase in overall survival (OS) and recurrence-free survival in several cancers, but this was also associated with great toxicity percentages [79]. Both checkpoint inhibitors (anti-CTLA-4 and the PD-1 antibodies) can cause pruritus in 18–34% of treated subjects [80], with differences between the latter two forms of therapy.

Yang et al. examined the occurrence of dermatologic adverse effects (AEs) and established the risk of these side effects due to PD-1/PD-L1 inhibitors, as compared to conventional chemotherapy or ipilimumab [81]. Pruritus and rash were the most described dermatologic AEs, with an incidence of 12.2% and 11.8%, respectively. Compared with neoplastic subjects getting chemotherapy, PD-1/PD-L1 inhibitor-treated subjects presented a greater risk of developing pruritus, and rash; however, anti-PD1/PD-L1 treatment presented a lesser risk of provoking pruritus with respect to ipilimumab. This report states that anti-PD-1/PD-L1 drugs have a different dermatological security profile with respect to conventional therapy and anti-CTLA-4 treatment [81].

One study seems to confirm the risk of cutaneous side events in melanoma subjects treated with checkpoint inhibitors [82]. Cutaneous side effects of any grade happen in about 30% of subjects, and side effects of grade 3, 4, or 5 happen in up to 10% of patients [83]; similar findings were reported by several other studies [84,85,86,87,88,89,90,91,92,93]. These data were also validated by a recent report that studied a cohort of 285 immune-related adverse events (irAEs) subjects with various malignancies, reporting that pruritus (34%) and maculopapular rash (28%) were the dominant cutaneous ICI-related toxicities. Moreover, the authors evaluated irAEs’ histological pattern distinguished by perivascular/interface inflammatory lymphocytic/eosinophilic infiltrate. Grave irAEs presented augmented concentrations of IL-6, IL-10, and eosinophilia, implying multifactorial pathogenesis [50].

Finally, new combined treatments with ICIs have been recently investigated and the onset of skin events has been analyzed. Doi et al. evaluated the security and effectiveness of combined mogamulizumab, a new anti-CCR4 antibody, and nivolumab in immunotherapy-naive subjects with metastatic cancers. The most commonly reported adverse effects were pruritus (11%), rash (39%), and maculopapular rash (20%) [94].

Patients with untreated stage IIIB/IV EGFR-mutant non-small cell lung cancer (NSCLC) were treated with pembrolizumab plus erlotinib. In this study too, the most reported treatment-related side effects with pembrolizumab plus erlotinib were pruritus (33.3%) and rash (50.0%) [52].

Generally, from a clinical point of view, pruritus appears at the start of treatment and may continue for numerous months after the suspension of therapy. The torso and extremities are the places most affected by pruritus, followed by the acral areas, neck, and head [95].

A genetic susceptibility could be relevant for the occurrence of pruritus and skin alterations. In a report, subjects with advanced melanoma were cured with anti-PD-1 treatment, and 22% of subjects presented cutaneous reactions [51]. Gene expression analysis of skin demonstrated a gene expression profile with an increase of several cytotoxic mediators such as perforin 1 (PRF1) and granzyme B (GZMB), inflammatory chemokines, such as CXCL9, CXCL10, and CXCL11, and the pro-apoptotic molecule Fas ligand (FASLG), as well as an increase of PD-L1. Moreover, the expression profile of specific genes in the skin alterations was different from that seen in skin alterations due to other treatments. The gravity of the immune-mediated injury varies and is interindividual, and a possible justification could be the genetic inclination based on single nucleotide polymorphisms (SNPs) in genes correlated to immune functions. These genetic background modifications can provoke changes in the predisposition to get cutaneous drug reactions [51].

A remarkable aspect of some reports is the presence of a probable correlation between irAEs and treatment effectiveness in tumor subjects who were treated with nivolumab or pembrolizumab. Finding from these experimentations demonstrated that the occurrence of any grade of skin irAEs can be considered as a predictor of a better outcome. In fact, it was reported that subjects with skin irAEs had higher ORR, PFS, and overall survival than subjects without skin irAEs, particularly if these skin irAEs happened soon, within six weeks of drug administration [96,97,98,99,100,101].

## 4. Targeted Therapy and Pruritus

Recently, targeted anti-tumor treatments, comprising small-molecule tyrosine kinase inhibitors (TKIs) and monoclonal antibodies (mAbs), were employed for the therapy of lung, breast, colorectal, and several other tumors [102,103]. In contrast to the conventional anti-tumor chemotherapy that non-specifically harms tumor cells as well as growing normal cells, the novel targeted anti-tumor drugs selectively inhibit signal pathways correlated with specific tumor proliferation, and in doing so, efficaciously decrease systemic side effects [104]; however, targeted anti-tumor drugs often cause cutaneous alterations. Drug-provoked pruritus has even been reported to happen more commonly with targeted anti-tumor drugs than non-targeted agents [105,106].

Among the monoclonal antibodies, a fundamental action in cancer patients is performed by anti-CD30 antibodies. Subjects with primary refractory Hodgkin’s lymphoma have bad outcomes. Although several salvage protocols have been proposed, there is no standard of care. Children’s Oncology Group protocol AHOD1221 (NCT01780662) verified Brentuximab vedotin with gemcitabine in patients with primary refractory Hodgkin’s Lymphoma. The most common grade 3–4 collateral events were comprised of rash (36%) and pruritus (10%) [107].

Pruritus is also a frequent event with other targeted anti-tumor agents such as multikinase inhibitors (MKIs) (19%) and Bcr-Abl inhibitors (13%), although grade 3 pruritus is rare (<3%).

The MKI sorafenib is employed for the therapy of hepatocellular carcinoma and metastatic renal cell carcinoma. The most recurrent collateral effects comprise dermatologic alteration, which may happen in more than 10% of the treated subjects [108].

Imatinib mesylate is a small-molecule TKi created to target c-ABL and BCR-ABL, employed for the therapy of chronic myeloid leukemia and gastrointestinal stromal tumors. Cutaneous imatinib-induced alterations are common, usually modest, and dose-dependent [109,110], although all grades of skin alterations have been described, extending from pruritus to Stevens–Johnson syndrome [111,112,113,114].

As for the pathogenesis of cutaneous alterations happening during imatinib treatment, a direct action of the tyrosine kinase block on the Platelet-Derived Growth Factor (PDGF) receptor, present on dermal mast cells, was proposed [37]. The inhibition of this receptor might provoke an increase of dermal interstitial fluid pressure with the consequent onset of cutaneous edema and erythema; however, the histological proof for an increased number of dermal mast cells, which present a functional c-kit receptor, in patients with grave cutaneous alterations from imatinib mesylate appears to reject a direct action of the imatinib on mast cells [115,116].

Imatinib mesylate-related dermatologic alterations might be also correlated to the delivery of IL-31 and IL-33. The release of IL-33 and the subsequent interaction with its receptor on mast cells causes the production of numerous substances capable of provoking skin alterations, comprising IL-31, a known pruritus-inducing cytokine [38].

The employment of other targeted therapies, and in particular Epidermal Growth Factor Receptor Inhibitors (EGFRIs), is also correlated with the onset of dermatologic toxicities, which provoke distress and negatively modify adherence with EGFRI treatment [117].

Epidermal Growth Factor Receptor (EGFR) belongs to the Erb group of tyrosine kinase receptors. The EGFR receptor family comprises four membrane receptors with tyrosine kinase activity: EGFR (ErbB1, Her1), ErbB2 (Her2), ErbB3 (Her3), and ErbB4 (Her4) [118,119,120]. EGFR has a fundamental effect in several physiological processes, and its principal regulators are Epidermal Growth Factor and Transforming Growth Factor-α.

An increase of these receptors is reported in several tumors, comprising colorectal, breast, malignant head and neck neoplasms, non-small-cell lung cancer, prostate, ovarian, cervical, stomach, and pancreatic cancer [121], and in clinical activity, substances that modify the function of EGFR are progressively more utilized. Among them we can find EGFR-inhibiting monoclonal antibodies such as cetuximab and panitumumab; EGFR tyrosine kinase inhibitors of the first-generation such as gerlotinib and efitinib; inhibitors of the second-generation such as trastuzumab, necitumumab, and dacomitinib; inhibitors of the third-generation such as pertuzumab, sapitinib, osimertinib, varlitinib, rociletinib, olmutinib, poziotinib, and vandetanib. Finally, we have multitarget tyrosine kinases inhibitors such as canertinib, afatinib, lapatinib, and neratinib [122,123,124,125,126].

The inhibition of EGFR function considerably alters epidermal homeostasis. The mechanism of EGFRI-provoked dermatologic alterations is to block the normal proliferation and differentiation of the epidermis, leading to dermatologic symptoms such as pruritus, xerosis, and inflammation [39,40]. Moreover, EGFR stimulation controls epidermal growth by blocking keratinocyte programmed cell death [41,42]. Activation of the receptor is correlated with the diffusion of a signal able to induce the transference of keratinocyte from the G1 phase to the S phase of the cell cycle [127]. Epidermal Growth Factor (EGF) also modifies the growth of sebaceous and sweat glands [128].

In an experimental animal model, the pharmacological block of EGFR is correlated with aggravation of skin inflammatory conditions and increased production of chemokines in keratinocytes [43,44,45]. In vitro and in vivo studies performed in patients confirmed that blocking EGFR signaling increased inflammation in human keratinocytes [129] and clinical suggestions indicate that the local cutaneous response could have systemic repercussions, as we can also have modifications in circulating chemokines and cytokines in treated patients [130,131,132,133]; however, it is unknown what actions have classical mediators, such as histamine and neurotransmitters, comprising serotonin, opioids, and γ-aminobutyric acid [134].

Pruritus arises among 57% of subjects treated with panitumumab, and 13% of those treated with erlotinib [135]. During therapy, systemic or local pruritus was reported, varying in strength from mild to severe itching. It often co-occurs with xerosis and papulopustular rash. Pruritus can happen with or without visible skin changes; in some patients, there are no observable skin alterations other than xerosis. Clinical reports stated xerosis percentages of 7–90% and pruritus percentages of 15–60% under EGFRIs, with augmenting percentages in the long-term treatment of up to 100% for both pruritus and xerosis in subjects treated at least for six months [136,137,138,139].

Other reports have described the dermatological side effects of cetuximab, a different specific inhibitor of EGFR. Cetuximab, dispensed in combination with conventional chemotherapy, has been demonstrated to increase chemotherapy effectiveness in tumors of the head and neck and metastatic colorectal cancer [140,141]; however, cutaneous alterations are the main collateral effects correlated with cetuximab administration and they comprise of pruritus [117,142].

Analogously, the administration of panitumumab in patients affected by advanced colorectal cancer has ameliorated prognosis and overall survival but is often correlated with cutaneous alterations, the most frequent of which are pruritus and papulopustular rash [143], and its utilization has been impeded by the presence of dermatological toxicities in the greater part of treated subjects (>90%) [144,145].

The ability of target therapy to induce pruritus was validated by an investigation directed to evaluate the features of pruritus, employing a questionnaire-based analysis. A total of 374 tumor patients took the survey, of which, 108 were administered with the targeted treatment. A total of 205 subjects had pruritus, of whom, 66 were under the targeted therapy. EGFRI-treated patients presented the greatest incidence of pruritus and highest numeric evaluation scale score for itching [146].

A relevant aspect of the cutaneous alterations provoked by EGFR inhibitors is the chance that they can require an interruption in anti-tumor treatment; however, there is a complete accord that suspension of anti-EGFR treatment-based therapy should be avoided [147]. All guidelines report the suggestion that subjects with cutaneous alterations should be examined by a dermatologist and that resolution of treatment should be founded on the nature and the gravity of the dermatological events [147,148,149,150].

In addition to conventional chemotherapy and target therapy, pruritus could be induced by other forms of cancer treatment such as radiotherapy. Collateral effects of radiation-treatment, often combined with chemotherapy or targeted therapies, on the skin comprise acute and chronic inflammation with pruritus and pain, and these may modify patients’ quality of life. Relevant acute cutaneous toxicities may involve up to 95% of these subjects [151]. New radiation techniques enable the skin to be exposed to a minimal amount of radiation that is directed specifically at the intended target, but in some specific cases, such as head and neck cancer, the skin is so near target volumes that the onset of inflammation cannot be prevented. Moreover, the combination of radio-sensitizing systemic therapies (e.g., 5-fluorouracil, Cetuximab, etc.) augments this toxicity [152,153,154,155].

Finally, even the transplantation approach in the therapy of tumors can induce itching. Allo-hematopoietic stem-cell transplantation (HSCT) patients are subjected to composite drug protocols, augmenting the risk for pruritus [156]. A report evaluated the possible causes of skin alterations in the first year after CD34+-selected peripheral blood stem cell transplantation (PBSCT) [157]. A retrospective study evaluated 243 subjects who experienced CD34+-selected PBSCT. A total of 152 patients (63%) presented rash within 1 year after PBSCT. The percentage of subjects with pruritus was not different between those with an acute Graft-versus-Host Disease (aGVHD) rash versus non-aGVHD rash. The most frequent reason for non-aGVHD rash was drug-related. Single drug culprits were recognized in 51% of rashes. The most frequently described drugs comprised chemotherapy, antibiotics, keratinocyte growth factor, and recombinant IL-7 [157].

## 5. Conclusions

Pruritus is an underestimated problem in patients living with cancer. Cutaneous alterations due to chemotherapy and chemoradiation are very frequent and may provoke even life-threatening complications augmenting morbidity and mortality. It should also be observed that some cutaneous collateral effects that occur during therapy, such as pruritus, can affect poor adherence to the therapy protocol or cause the need for dosage changes or even discontinuation of treatment.

Although we could imagine that pruritus might be more of a problem in subjects on lower-dose, long-term treatments with conventional chemotherapy, a clinically relevant amount of pruritus may be observed among patients getting new treatments such as targeted therapy [158].

Several new drugs can be responsible for the onset of acute or chronic itch; however, the causal mechanisms have not yet been investigated in depth, and different mediators could be implicated in the pathology of this symptom. Further exhaustive studies on the incidence of drug-caused pruritus after the employment of specific new medications, as well as exploration of its pathomechanisms, are urgently needed. In the near future, data derived from these investigations will permit changes in the treatment of this sort of pruritus, and itchy conditions will be considered and treated by targeting specific pathways at their biomolecular level. The identification of specific humoral mediators will help identify possible new drugs to treat this condition. All this will make it possible to make the best use of the new antineoplastic drugs, optimizing their effectiveness and reducing possible skin side effects.

Moreover, data about the treatment of drug-provoked cutaneous reactions of antineoplastic agents emphasize the value of interdisciplinary cooperation between oncologists and dermatologists. The complete knowledge of dermatological collateral effects and their appropriate management are essential to improve patients’ quality of life and guarantee sustainability of oncological therapies. Creation of multidisciplinary teams is crucial for optimal management of these drugs.

Finally, with respect to drugs, studies on the relationship between the onset of skin alterations and outcomes, and between the onset and severity of skin lesions and genetic predisposition [159,160], could result in new perspectives on the treatment of neoplasms through specific therapy.

## Figures and Tables

**Table 1:** Possible mechanisms and characteristics of antineoplastic drug-induced pruritus.

Drug	Mechanisms	Characteristics	Ref.
**Paclitaxel**	Skin lesions	Acute pruritus	[23,24,25,26]
**Nab-paclitaxel**	Skin lesions induced by albumin constituents		[24,25,27,28,29]
**Chlorambucil**		Delayed reaction	[30]
**Cytarabine**	Immune reactions Epithelial toxicity Changes in keratinocyte activities		[31,32,33,34,35,36]
**Imatinib**	Inhibition of PDGF receptor on dermal mast cells Release of Il-33 and IL-31		[37] [38]
**Epidermal Growth Factor Receptor inhibitors**	Alteration of epidermal homeostasis Effect on keratinocyte apoptosis Increased release of chemokines	Acute pruritus	[39,40] [41,42] [43,44,45]
**Erlotinib**	Increase of mast cells Changes in keratinocyte activities Release of IL-1, TNF, IL-8	Acute pruritus	[46] [39] [47,48]
**Lapatinib**	Unidentified mechanism	Acute pruritus	[49]
**Immune checkpoint inhibitors**	Release of inflammatory cytokine (IL6, IL-10)	Acute pruritus	[50]
**Anti-PD1**	Release of perforin1, granzyme B, CXCL9, CXCL10, CXCL11		[51]
**Pembrolizumab**		Chronic pruritus	[52]
